# Hay Fever

**Published:** 1890-09

**Authors:** 


BUFFALO MEDICAL AND SURGICAL JOURNAL

A MONTHLY REVIEW OF MEDICINE AND SURGERY.

*EDITORS:*

THOMAS LOTHROP, M. D. - - WM. WARREN POTTER, M. D.

All communications, whether of a literary or business character, should be addressed to the editors : 284 Franklin Street, Buffalo, N. Y.

Vol. XXX. SEPTEMBER, 1890. No. 2.

### Editorial.

### HAY FEVER.

The season of this perennial malady is upon us, and we are glad to offer our readers some late thoughts on the subject in this issue of the Journal, which will be found in the proceedings of the Buffalo Medical and Surgical Association.

Dr. William H. Daly, of Pittsburgh, who has long been recognized as competent authority on laryngological questions, has also done much to increase our knowledge in regard to hay asthma. Dr. Daly was chosen to deliver the annual address on Laryngology before the Medical Society of the State of Pennsylvania, at its meeting held in Pittsburgh in June of the present year, and we select that portion of his address published in the *Medical Mirror*, July, 1890, pertaining to this disease, which will prove of interest in connection with the discussion above alluded to as published elsewhere in this number.

In discoursing upon hay asthma, says Dr. Daly :

Concerning what laryngology and rhinology have done in the successful treatment of hay asthma, I probably feel myself more competent to speak than upon some other questions. Since 1881, when I first made known to the profession my observations upon hay asthma, in a paper which I read before the American Laryngological Association, I have treated over two hundred cases of this very troublesome disease ; but it is of the first two hundred cases that I shall now speak. I have taken pains in the treatment of all these cases of hay asthma, to confine myself, as far as possible, to the treatment of putting the intra-nasal tissues in a normal and healthy condition ; whether by means of surgical operative procedures, or by the milder methods of local medicinal applications. This has resulted in a cure of sixty-eight per cent. of these cases, and with a large portion of the remaining thirty-two per cent. more or less improved, although proper subjects for more thorough treatment, with fair prospects of further benefit or cure. In asthma, the results have been also satisfactory, at least far more so than by any method which does not include the restoration of the intra-nasal and naso-pharyngeal structures to a normal and healthy condition.

In one hundred cases of asthma the cures have been thirty-four per cent., with a large proportion of the remaining cases benefited. There is something strange, indeed, in the way in which the subjects of hay asthma deny themselves the rational investigation of their disease, hugging the old and threadbare theories of hay asthma and its tentative treatment to their bosoms, and going on from Summer to Summer, looking forward to the recurrence of their attack with a sort of valetudinarian glee, that upon such a date they will enter upon a new sort of life, so to speak, when they will have the doleful pleasure of sneezing and sniffling and growling. And if the patient be a medical man,—the worst sort of a patient, by the way,—he not only wants to be considered a very sick patient, but wants to "boss the job" while his professional brother endeavors to cure him. When a medical man finds himself in the midst of his recurrent hay asthma, with the upper air passages dammed so as to make his breathing a difficult and disagreeable matter, unless he be a very humble and Christian man, he in turn "damns" everything that comes within his reach, whether it be domestic or professional, and will not hesitate to "damn" his professional brother too, whom he calls upon for relief, if he is not handled as tenderly as if he were a sick kitten. I speak from experience with my professional brothers suffering with hay asthma. But let the doctor once get over his acute attack and he would not let you touch the interior of his nose for the world; he is too afraid of getting hurt.

Now as to the clergyman with hay asthma: he knows when he has a sure thing that will get him a trip, annually, to the Adirondacks, where he can shoot deer, with young fawns at their side, out of season; or make a trip across the ocean, on funds from a fat subscription, started by some of the adoring old maids of the parish. This clergyman don't want his nose looked into for any cause of hay asthma, "not he"; that nose is his best friend; it is like the "blind eyes" to the beggar, or the "wooden leg" to the candidate for alms; it is his capital stock that annually turns loose as dividends the golden flood gates of sympathy—gets him "a rest," and also his congregation "a rest."

Now, gentleman, I hope I may not be accused of taking too great liberties with either the status of the medical or clerical professions, upon the question of hay asthma, when I say I believe they deserve all the animadversion I have indulged in, and more too. And as to the respectable and exclusive society of hay asthma sufferers, who annually congregate in various locations of New England to shed tears and sneeze over one another's shoulders, and in one another's faces, in a sort of "vaso-motorio rhinological" love feast, where the congregations, employers, and others not directly interested, help pay for the entertainment; I have had members of this society shake their heads at me and say, "You doctors cannot cure my hay asthma; nobody can cure it; it cannot be cured." To which I have sometimes replied: "Nor do you want to try to have it cured, by the only rational method known to modern medicine." And I have often been correct in my opinion, for these men would not have the inside of their noses cut, or burned, or sawed for anything; it might hurt. Some of them — nay, the most of them — have never even had the interior of their noses examined by a medical man competent to discover abnormalities, and cure them when he finds them.

Now I may ask in the same spirit that the christian believer asks the propagator of the doctrine of infidelity, What better have you to offer me than my faith and practice which I now hold? What results can compare with those I have given you in the treatment of hay asthma? and what are the carpings of the unbeliever, when he is unable to say, "I have no surer scheme of salvation to offer you if you relinquish the one you are now adapting to the benefit and relief of suffering humanity."

